# Safety Assessment of Zigbir®: A Polyherbal Formulation in Sprague-Dawley Rats

**DOI:** 10.1155/2012/589520

**Published:** 2012-10-18

**Authors:** Joseph Joshua Allan, Ranjit Madhukar Bhide, Amit Agarwal

**Affiliations:** ^1^R&D Centre, Natural Remedies, Plot No. 5B, Veerasandra Industrial Area, 19th K.M. Stone, Hosur Road, Electronic City, Bangalore, Karnataka 560 100, India; ^2^Indian Institute of Toxicology, 32/A/01, Hadapsar Industrial Estate, Pune, Maharashtra 411 013, India

## Abstract

The safety of Zigbir®, a polyherbal formulation intended for use as food supplement, was evaluated in Sprague-Dawley rats treated orally at the dose of 2000 mg/kg in acute and at 250, 500, and 1000 mg/kg for 90 days in subchronic toxicity study. The median lethal dose of Zigbir® was found to be more than 2000 mg/kg, and fourteen-day repeated dose toxicity study revealed it to be safe up to 1000 mg/kg. The subchronic study did not show any mortality or treatment-related adverse clinical signs. The treated animals exhibited normal feed intake and comparable body weight gain except for a decrease in females of 500 and 1000 mg/kg groups. Ocular examination revealed no abnormalities. Further, Zigbir® administration in rats did not induce any major changes in urinalysis, hematological, and biochemical evaluations except for minor alterations in few parameters at different dose levels. Gross and histopathological findings did not show any lesions attributable to Zigbir® administration. The no observed effect level of Zigbir® was found to be 500 and 250 mg/kg in male and female Sprague-Dawley rats.

## 1. Introduction

Development and assessment of herbal formulations for various beneficial health and or functional effects in animal and human species are increasing in popularity. Despite well-established scientific studies on phytopreparations or herbal ingredients for pharmacological properties, the safety details of herbal substances reported in compliance to internationally accepted guidelines seem to be inadequate [1–3]. In view of anticipated increase in use of herbal supplementation in future for various health needs, short-and long-term toxicological investigations are required for evaluation and classification of herbal preparations based on safety data. 

The investigational substance of the present study, Zigbir®, is a polyherbal formulation consisting of four medicinal plants, namely, *Andrographis paniculata *(*A. paniculata*), *Boerhaavia diffusa *(*B. diffusa*), *Phyllanthus amarus *(*P. amarus*), and *Solanum nigrum *(*S. nigrum*) that protect the liver from variety of toxins. This polyherbal preparation, recommended in poultry birds, tones up the liver and enhances hepatic functions, accelerates the regeneration process, and stimulates sluggish liver parenchyma. It also improves the secretion and flow of bile and helps in fat metabolism. 

The hepatoprotective effects of herbal ingredients of Zigbir® have been well established in published literature. *A. paniculata* (Acanthaceae), also known as King of Bitters, is extensively used alone or in combination with other herbs in many hepatoprotective preparations in Indian system of medicine [4–6]. Andrographolide, the major active constituent of *A. paniculata* and a diterpenoid lactone, has been shown to be chiefly responsible for the antihepatotoxic activity of the herb in different animal models. Andrographolide showed protective effect against carbon tetrachloride [7], paracetamol [8], ethanol [9], and galactosamine [10] induced hepatotoxicity in experimental animals. A study by Chaudhuri revealed that *A. paniculata* extract treatment increased biliary flow and liver weight and reduced the duration of action of hexabarbital in laboratory rats [11]. Administration of other diterpenes such as andrographiside and neoandrographolide to mice caused a marked increase in cellular antioxidant components with a concurrent decrease in lipid peroxidation. The hepatoprotective properties were found to be equivalent to the beneficial effects of silymarin on liver functions [4].

An alcoholic extract of *B. diffusa *(Nyctaginaceae) on oral administration at 500 mg/kg to adult male Swiss mice and Charles Foster rats exhibited hepatoprotective properties, decreased barbiturate sleeping time, prothrombin time, and lowered plasma bilirubin levels. The extract was also reported to be safe even up to 2000 mg/kg in mice [12]. Chakraborti and Handa demonstrated the antihepatotoxicity activity of chloroform and methanolic extracts of *B. diffusa *against carbon tetrachloride-induced hepatic damage in isolated rat hepatocytes [13].

*P. amarus* (Euphorbiaceae) at 200 mg/kg treated orally improved liver regeneration in male Wistar rats with alcohol induced liver damage [14]. Mehrotra et al. investigated the *in vitro *effect of *P. amarus* on hepatitis B virus and found that the alcoholic extracts of *P. amarus* were effective against HBV antigens, the butanol extract being the most potent [15]. The active fractions inhibited the interaction between HBsAg/HBeAg and the related antibodies indicating the anti-HBs, anti-HBe-like activity and also an effect on HBV-DNA. Under *in vitro* conditions, aqueous extract of *S. nigrum* (Solanaceae) suppressed aflatoxin production by *Aspergillus flavus* and thereby protected the liver. The hepatoprotective activity of *S. nigrum* could be due to the inhibition of oxidative degeneration of tissue DNA [16].

Despite the availability of extensive pharmacological information for the aforementioned medicinal plants on the hepatic function, only few reports are existing that describe the safety evaluation of individual plant ingredients carried out in conformity to universally accept testing protocols. In addition to the beneficial effects observed, it is also imperative to generate comprehensive toxicological information of the test substance for ensuring safety upon use, especially for longer periods. In the present study, therefore, we conducted acute and subchronic oral toxicity studies on Zigbir® in rats, to establish the no observed effect level (NOEL) and to determine its safety to be used as a hepatoprotective agent.

## 2. Materials and Methods

### 2.1. Test Substance

Zigbir® is a combination of four medicinal herbs developed by M/s Natural Remedies, Bangalore, India. Zigbir® has *A. paniculata* (27.7% w/w), *P. amarus* (27.7% w/w), *S. nigrum* (27.7% w/w), and *B*. *diffusa* (16.9% w/w). The plant materials used were analyzed for respective marker compounds by high performance liquid chromatography (HPLC) method and by liquid chromatography-mass spectrometry (LCMSMS) methods.

#### 2.1.1. Standardization of Zigbir®

The crude powders obtained from the plant materials, after verifying the content of marker compounds, were mixed in appropriate proportions, grinded, and blended to prepare Zigbir®. The thin layer chromatography (TLC) profile of product was compared with the reference material using high performance thin layer chromatography (HPTLC) analysis. The product and respective reference standard weighing 5.0 g were weighed separately and extracted separately using reflux condenser on a water bath using 80 mL methanol for 30 min. The filtrate is decanted and the residue is again extracted with 80 mL methanol for 30 min and the process is repeated twice and then filtered after cooling. The filtrate was concentrated to 25 mL. Equal volumes (15 mcg) of sample and reference standard were spotted on Silica gel 60 F_254_ plate of 0.2 mm thickness as bands. The plate was developed in a mobile phase consisting of toluene : ethyl acetate : acetic acid (55 : 45 : 2). The dried plate was scanned at 254 and 366 nm. The plate was sprayed with anisaldehyde sulphuric acid reagent and dried in oven at 100°C. The fingerprint of the product sample was compared with reference standard.

### 2.2. Experimental Animals and Housing

Sprague-Dawley rats (6–8 weeks) used in the present studies were bred and reared at Indian Institute of Toxicology, Pune, India. The animals were housed in polypropylene cages with stainless steel grill tops and bedding of clean paddy husk in a temperature-controlled animal room (20–24°C) with a relative humidity of 30–70%, 10–15 air changes per hour, and illumination cycle set to 12 h light and 12 h dark. Standard pelleted rodent feed (M/s Nav Maharashtra Chakan Oil Mills Ltd., Pune) and filtered and UV exposed portable water (Aqua guard) were provided *ad libitum*.

### 2.3. Acute Oral Toxicity Study

A preliminary sighting study, followed by main study, was conducted to assess the acute oral toxicity of Zigbir®, administered as a suspension in 0.5% aqueous carboxymethylcellulose (CMC) to five female rats (eight weeks old) at 2000 mg/kg body weight as a single oral dose in a sequential manner. The dose volume was kept as 20 mL/kg body weight. The rats were fasted 16 h before and 3 h after the administration of the test material and only water was provided during the period. All the animals were observed for mortality (twice daily) and clinical signs for first 10 min, 30 min, 1 h, 2 h, 4 h, and 6 h after dosing and thereafter once a day for continuous 14 days. The body weight of rats was recorded following the period of fasting on day 0, weekly thereafter and at termination of study on day 15 and weekly body weight gain was calculated. Necropsy was carried out on all rats that were sacrificed at the end of the experiment. This study was performed in compliance with the OECD guideline for testing of chemicals: Test guideline No. 420, Acute oral toxicity-fixed dose method and with Schedule Y, Drug and Cosmetics (Eighth amendment) Rules-1988, Ministry of Health & Family Welfare, Government of India [17, 18]. 

### 2.4. 14-Day Dose Range Finding Study

A dose range finding study was conducted to evaluate the potential toxicity of repeated exposure of Zigbir® in Sprague-Dawley rats to select dose levels for a 90-day subchronic toxicity study. In this study, groups of 5 male and 5 female rats were administered freshly prepared doses of Zigbir® daily by oral gavage for a period of 14 days at 100, 250, 500, and 1000 mg/kg. A concurrent control group of rats received vehicle (0.5% CMC) alone. The rats were observed for mortality and clinical signs during the 14-day observation period. Body weight and feed intake of treated rats were recorded at initiation of the study and weekly thereafter. All the surviving rats were sacrificed on day 15 for gross pathological examination.

### 2.5. Subchronic Oral Toxicity Study

#### 2.5.1. Doses and Treatments

A 90-day, subchronic oral toxicity study was conducted in accordance with the OECD guideline for testing of chemicals: Test guideline No. 408, repeated dose 90-day oral toxicity study in rodents [19], and in compliance to Schedule Y, Drug and Cosmetics (Second amendment) Rules-2005, Ministry of Health & Family Welfare, Government of India to evaluate the toxic potential of Zigbir® on repeated oral exposure [20]. Fifty each male and female Sprague-Dawley rats were selected for the study. The animals were randomly allotted into four groups each consisting of 10 male and 10 female rats (6 to 8 weeks old). The animals were administered orally with Zigbir® (in 0.5% CMC) at the dose levels of 0, 250, 500, and 1000 mg/kg once daily for consecutive 90 days. Additional satellite groups for control and high dose level, having five rats per sex, were given respective treatments for 90 days and were kept for further 28 days to assess for reversibility, persistence, or delayed occurrence of toxic effects, if any. The females were nulliparous and nonpregnant. The dose volume was kept constant at 10 mL/kg for all dose levels including the control group and the dose volume administered to individual rat was adjusted according to its most recently recorded body weight. The doses were prepared fresh daily, before administration. The rats were dosed at approximately the same time each day. 

#### 2.5.2. Mortality and Clinical Signs

All animals were observed twice daily for mortality during the study period. Also, abnormal clinical signs or reactions to treatment (time of onset, intensity, and duration), if any, were recorded. 

#### 2.5.3. Body Weight and Feed Intake

The body weight and feed consumption data were recorded on the day of commencement and at weekly intervals throughout the study period in all the groups and in recovery groups during the posttreatment period also.

#### 2.5.4. Ophthalmoscopy

Eye examination of control and all the treated animals was conducted prior to initiation of the study and during weeks 13 and 17 (for recovery groups), using a hand slit lamp after induction of mydriasis with 0.5% tropicamide solution.

#### 2.5.5. Functional Observations

During week 13, all animals were examined for sensory reactivity to auditory, visual, and proprioceptive stimuli, grip strength (Digital grip strength meter, Columbus), and motor activity. A functional observational battery was designed to evaluate the response of the animals in home cage and open field conditions.

#### 2.5.6. Clinical Pathology

At the end of week 13 (control and treated groups) and week 17 (recovery groups), all rats were fasted overnight. Blood samples were collected from retroorbital sinus under ether anesthesia for haematology and blood biochemistry using potassium EDTA (1.5 mg/mL) and sodium heparin (200 IU/mL) as anticoagulants, respectively.

Haematological parameters estimated (Beckman Coulter Analyzer Ac.T.Diff, Source: Wipro Biomed Ltd., Mumbai, India) included haemoglobin (Hb), erythrocyte count (RBC), reticulocyte count (Rt), haematocrit (HCT), mean corpuscular volume (MCV), mean corpuscular haemoglobin (MCH), mean corpuscular haemoglobin concentration (MCHC), platelet count (PLT), and total white blood cells (WBCs). Differential leukocyte counts were performed manually from microscopic specimens. Prothrombin time (Pt) was measured using citrate bulb (100 *μ*L of 3.8% solution of sodium citrate per mL of blood). Blood samples were centrifuged at 3000 rpm for 15 min.

The serum biochemistry parameters studied (VeTEX Veterinary Chemistry Expert, Source: Wipro Biomed Ltd., Mumbai, India) were alanine aminotransferase (ALT), aspartate aminotransferase (AST), alkaline phosphatase (ALP), gamma glutamyl transferase (*γ*GT), lactate dehydrogenase (LDH), bilirubin, blood urea nitrogen (BUN), creatinine, total protein, blood glucose, creatinine phosphokinase (CPK), total cholesterol, triglycerides, albumin, calcium, inorganic phosphorous, sodium, potassium, and chloride.

#### 2.5.7. Urinalysis

The urine samples were collected on day 91 from control and treated groups and on day 119 from recovery groups to analyse urine volume, appearance, colour, pH, specific gravity, proteins, glucose, ketones, occult blood, bilirubin, urobilinogen, and nitrite. Microscopical examinations were conducted for presence of pus cells, epithelial cells, casts, RBC, and crystals and frequency of the analytes, if present, were recorded. 

#### 2.5.8. Necropsy and Histopathology

All surviving rats were sacrificed at termination of the study by exsanguination under carbon dioxide anesthesia and were subjected to complete necropsy. The absolute weights of organs such as liver, kidneys, adrenals, spleen, lung, brain, heart, uterus, and testes/ovaries were recorded and the relative weights (i.e., organ/body weight ratios) were calculated. The following organs and tissues were preserved in 10% formalin and were subjected to histopathological examinations: adrenals, aorta, brain, caecum, colon, duodenum, epididymis, heart, ileum, jejunum, kidneys, liver, lungs, lymph nodes, oesophagus, ovaries, pancreas, pituitary, prostate, rectum, salivary glands, sciatic nerve, spinal cord, skeletal muscle, spleen, sternum with bone marrow, stomach, seminal vesicles, testes, thymus, thyroid, trachea, urinary bladder, and uterus. 

#### 2.5.9. Statistical Analysis

The data were evaluated by analysis of variance followed by Student's *t*-test, Cochran *t*-test, and Dunnett's test. Statistical significance was set at *P* < 0.01–0.05. Histopathological observations were tabulated and individual animal score was calculated according to degree and area (LABCAT Module for Histopathology, Innovative Programming Associates Inc., Princeton, New Jersey).

## 3. Results

### 3.1. Acute Oral Toxicity Study

All animals administered with a single oral dose of 2000 mg/kg of Zigbir® survived throughout the experimental period and did not show any signs of toxicity immediately following dosing and during the observation period of 14 days. The findings of the study did not reveal any major adverse effect on the body weight gain throughout the treatment period. The body weight gain after 7 and 14 days was found to be 16.54% and 28.55% in sighting study and 14.63% and 29.98% in main study, respectively. No major gross pathological changes were observed on necropsy. Based on the results, the median lethal dose for Zigbir® was found to be greater than 2000 mg/kg body weight.

### 3.2. 14-Day Dose Range Finding Study

All the male and female animals from control and different dose levels of Zigbir® survived till the terminal sacrifice and no abnormal clinical signs were noticed throughout the 14-day study period. There were no differences in body weight gain between the control and the treated group rats. Necropsy examination did not show any treatment-related evidence of toxicity.

### 3.3. Subchronic Oral Toxicity Study

#### 3.3.1. Mortality and Clinical Signs

Treatment with Zigbir® at selected doses produced no deaths, adverse clinical signs, or toxic effects in the animals throughout the dosing period of 90 days and the postdosing recovery period of 28 days.

#### 3.3.2. Body Weight and Feed Intake

**Figure 1:**
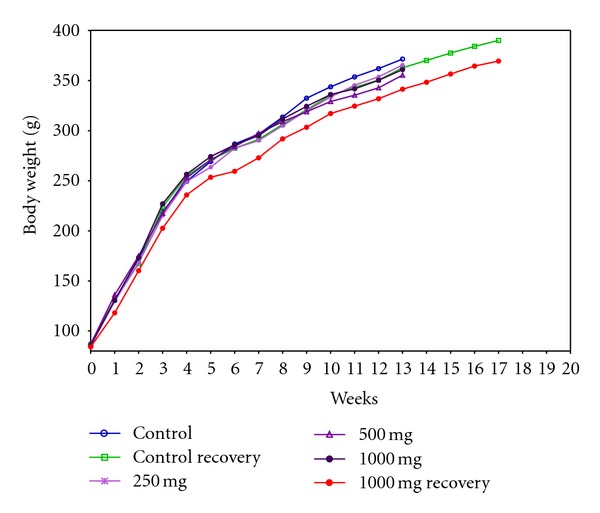
Group mean body weight of male rats orally administered with Zigbir® for 90 days.

**Figure 2:**
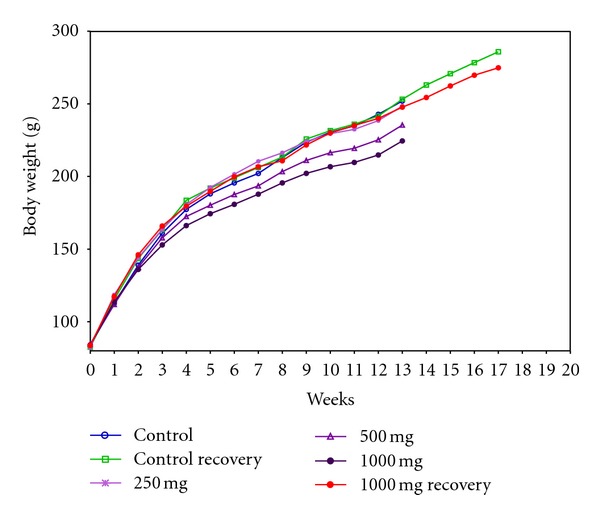
Group mean body weight of female rats orally administered with Zigbir® for 90 days.

During the complete experimental period, male animals from all the treated dose groups and recovery groups exhibited comparable body weight gain with that of respective controls (Figure 1). A reduced body weight gain of 6.56% and 10.90% was observed in female animals from 500 and 1000 mg/kg dose groups when compared with controls at the end of the study period of 90 days. Females from high dose (1000 mg/kg) recovery group exhibited normal body weight gain during the recovery period of 28 days (Figure 2). No differences were observed with respect to feed consumption in all the treated groups at various time intervals of evaluation (Table 1).

#### 3.3.3. Ophthalmoscope Examination

Ophthalmoscopic examination of control and all the treated dose group rats did not show any abnormalities.

#### 3.3.4. Haematological Investigations

Evaluation of haematological parameters revealed only few statistically significant decreases (MCV, males in the 250 mg/kg group; MCH and MCHC, males in the 250, 500, and 1000 mg/kg groups (*P* < 0.01); MCH, females in the 250 mg/kg group and MCHC, females in the 250, 500, and 1000 mg/kg groups (*P* < 0.01); HCT, females in 1000 mg/kg recovery dose group (*P* < 0.05) and increase (total RBC, in 250 mg/kg groups in males (*P* < 0.01) and females (*P* < 0.05); total WBC, males in 500 mg/kg group (*P* < 0.05); HCT, in 250 mg/kg in males and females and in females at 500 mg/kg (*P* < 0.01) and in females at 1000 mg/kg (*P* < 0.05), MCV in females at 500 mg/kg (*P* < 0.05) compared to control group (Tables 2 and 3).

#### 3.3.5. Serum Biochemistry

No treatment-related changes were observed from the blood biochemistry results except for elevated levels of alkaline phosphatase in animals from 1000 mg/kg dose group (*P* < 0.01) and decreased potassium levels in female rats of 250 mg/kg group (Tables 4 and 5).

#### 3.3.6. Urinalysis

There were no significant differences in any of the urinary parameters.

#### 3.3.7. Gross Pathology and Organ Weights

Treatment with Zigbir® did not induce any remarkable and treatment-related gross pathological alterations in treated animals. The groups mean absolute and relative weights of organs such as kidneys, liver, thymus, heart, testes, epididymides, ovaries, and uterus did not differ significantly from the control animals except for some changes in female animals, namely, increased relative weight (*P* < 0.01) of brain and spleen in animals of 250 mg/kg dose group and 500 mg/kg dose group, respectively. The 1000 mg/kg recovery group showed an increase in relative weight (*P* < 0.05) of adrenals (Tables 6, 7, 8, and 9).

#### 3.3.8. Histopathology

All the histopathological findings pertinent to this study appeared to be incidental as the frequency and severity remained identical for control and the treated animals of both sexes. The observations included leukocytic infiltration, necrosis in liver; leukocytic infiltration, hemorrhages in kidneys; alveolar histiocytosis, hemorrhages in lungs and gliosis, submeningeal, lymphocytic infiltration in brain.

## 4. Discussion

For several centuries, medicinal plants and herbal remedies continue to enrich the healthcare needs of animals and human. Liver, one of the essential organs involved in metabolism of xenobiotic substances and detoxification function in the body, is frequently challenged with numerous toxic assaults [21]. In the absence of effective and safe hepatoprotective agents in conventional treatment and because of prohibition of many antibiotics and synthetic growth promoters, medicinal herbs play important role in the management of various liver disorders [22, 23]. The beneficial effects of various medicinal plants are widely established in scientific literature and the formulations that contain single or multiple herbs have been indicated for restoring liver health in ethnomedical practices and traditional medicinal systems of many countries [24]. Poultry species are commonly and easily vulnerable to liver complications due to various etiological factors associated with commercial chicken production. The decreased liver performance in birds which eventually results in impaired metabolism, poor feed conversion efficiency, and decreased body weight gain that can be efficiently managed with herbal supplementation since herbs improves the threshold level of liver against different kinds of harmful stressors. However, herbal preparations, in spite of being popularly claimed as naturally safe, need to be authenticated by scientifically validated tests for toxicological properties before being introduced for widespread consumption.

In safety evaluation of test substances, acute oral toxicity study is considered as the preliminary step and facilitates classification and labeling of investigational agents [17]. In the present study, single acute oral administration of Zigbir® to five female Sprague-Dawley rats at the dose level of 2000 mg/kg b.w. did not cause any mortality and the median lethal dose was found to be more than 2000 mg/kg b.w. Therefore, the findings resulted in classifying Zigbir® in category 5 criteria according to the Globally Harmonised System. It could be observed from published scientific reports in recent times that many poultry and livestock herbal formulations made of polyherbal constituents akin to Zigbir® have been reported with the median lethal dose higher than 2000 mg/kg and found to be reasonably safe on acute exposure [1, 2, 25, 26].

Repeated dose oral toxicity studies are carried out to assess the adverse effects of a substance used for a prolonged period of time and to obtain information about the potential health hazards that may likely to occur from continuous exposure including information about target organ toxicity, possibilities of cumulative effects, and an estimate of the dose at which there is no observed adverse effect [19]. As commonly recommended by regulatory guidelines for subchronic toxicity testing, a 14-day dose range finding study was performed so as to select appropriate dose levels for the 90-day oral toxicity study. On administration of Zigbir® at 100, 250, 500, and 1000 mg/kg for a period of 14 days, no deaths, treatment-related abnormal clinical or behavioural signs, alterations in body weight gain, and gross pathological observations were recorded till the end of the experiment.

Based on the results of the dose range finding study, three proportionate dose levels of 250, 500, and 1000 mg/kg b.w. were selected for the subchronic oral toxicity study in rats. Treatment with Zigbir® orally for consecutive 90 days at and up to the dose level of 1000 mg/kg b.w. did not cause any mortality or toxicity signs during the dosing as well as postdosing recovery period in test groups. The results of the ophthalmoscopic and functional observation examinations of treated animals also confirmed the normal physiological responses that were similar to the findings of control group of animals. 

Guidelines of toxicity testing place considerable emphasis on reporting of changes in weight gain [17, 19]. For safety characterization of test materials, an accepted limit of 10% decrement in body weight or growth rate has been fixed under chronic exposure [27, 28]. In the current study, decreased weight gain was observed in female animals of mid and high dose groups at the end of the experiment. However, the male animals of respective dose groups and both sexes of treatment recovery groups showed normal body weight gain till the termination of the study. Despite the popular perception that xenobiotic compounds influence the body weight gain on prolonged ingestion, the inconsistencies noticed between the treated groups suggest that the alterations observed in body weight gain did not support apparent toxicological involvement. This consideration, as is evident from Table 1, is reinforced by the fact that the feed consumption of animals of all the dose groups remained unaffected throughout the study period and was found to be comparable with control animals at different weekly intervals.

On haematological analysis, some alterations in values of endpoints such as MCV, MCH, MCHC, HCT, total RBC, and total WBC were observed comparable to the control group. Additionally, on gross necropsy observation, there were no injuries to any of the haematopoietic organs or any alterations evident on histopathology. Though some alterations in hematological values were observed when compared to the control group, these variations cannot be considered as affirmative pathological changes of blood profile as no correlatable histopathological changes were observed. Moreover, the variations were found to be within limits and, hence, these changes were not considered as treatment related.

Evaluation of blood biochemistry revealed elevated levels of alkaline phosphatase in animals from 1000 mg/kg dose group. Though alkaline phosphatase is present in all tissues, the enzyme is particularly concentrated in liver, bile duct, kidney, bone, and placenta [29]. Hyperphosphatasemia, characterized by elevated alkaline phosphatase, is reported in conditions mainly of hepatic and bone diseases [30]. However, lack of concurrent, relevant macroscopic or microscopic evidences and absence of any such significant elevations in other related sensitive indicators of organ damage such as ALT, AST, *γ*GT, LDH, and bilirubin suggest that these findings may not be biologically significant. Decreased serum potassium levels were observed in males of low dose group alone and hence cannot be regarded as having any toxicological relevance. Also, the outcomes were correspondingly supported by findings of urinalysis.

One of the major advantages of conducting the subchronic toxicity studies is the information on specific organ toxicity upon repeated exposure of test substance intended for prolonged use in target animal species [19]. In this study, terminal sacrifice of various dose groups of Zigbir® did not reveal any gross pathological changes attributable to treatment. The absolute and relative organ weights of treated animals were found to be comparable with the respective control groups. Although few increases in relative weights of brain, spleen, and adrenals were noticed in some dose groups, these changes were found to be nondose dependent and hence considered to be of no toxicological importance. Similarly, the histopathological changes observed in the control and high dose treatment group animals were also comparable and can be considered either as incidental, congenital, or spontaneous. 

In addition to the *in vivo* findings of current study, recent *in vitro* toxicity testing on Zigbir® confirmed the relatively safe characteristics of herbal formulation in different test systems. Chandrasekaran et al. ascertained the *in vitro* genotoxicity potential of Zigbir® using Ames II assay and reported that the herbal substance did not exhibit any significant mutagenic effect on TA98 and TAMix strains of *Salmonella typhimurium* [31]. 

In conclusion, Zigbir® on single oral administration showed a median lethal dose of more than 2000 mg/kg b.w. in female Sprague-Dawley rats and did not exhibit any treatment-related clinical signs of toxicity when orally given continuously for 90 days at tested dose levels in both sexes of rats. The no observed effect level (NOEL) for Zigbir® in the subchronic oral toxicity study was found to be 500 mg/kg in male and 250 mg/kg in female Sprague-Dawley rats.

## Figures and Tables

**Table 1:** Group mean feed consumption of male and female rats given Zigbir® daily by gavage for 90 days.

Week	Control and control recovery		250 mg/kg				500 mg/kg				1000 mg/kg and 1000 mg/kg recovery			
	Male	Female	Male		Female		Male		Female		Male		Female	
0	8.20	6.98	8.25	101%	6.96	100%	7.92	97%	6.68	96%	8.19	100%	6.93	99%
1	11.40	10.21	11.25	99%	10.03	98%	10.85	95%	9.72	95%	10.76	94%	9.10	89%
2	12.22	11.05	12.02	98%	10.85	98%	11.62	95%	10.55	95%	11.57	95%	10.37	94%
3	13.06	11.82	12.89	99%	11.69	99%	12.46	95%	11.37	96%	12.45	95%	11.16	94%
4	13.74	12.60	13.69	100%	12.81	102%	13.27	97%	12.10	96%	13.22	96%	11.96	95%
5	14.64	13.74	14.47	99%	13.64	99%	14.01	96%	12.88	94%	14.06	96%	12.76	93%
6	15.42	14.48	15.21	99%	14.49	100%	14.81	96%	13.59	94%	14.83	96%	13.61	94%
7	16.22	15.27	16.06	99%	15.24	100%	15.67	97%	14.47	95%	15.60	96%	14.41	94%
8	17.03	16.07	16.87	99%	16.05	100%	16.45	97%	15.26	95%	16.41	96%	15.22	95%
9	17.83	16.98	17.69	99%	16.86	99%	17.21	97%	16.05	95%	17.22	97%	15.99	94%
10	18.57	17.68	18.44	99%	17.61	100%	18.05	97%	16.85	95%	18.02	97%	16.88	95%
11	19.45	18.51	19.21	99%	18.45	100%	18.86	97%	17.63	95%	18.80	97%	17.63	95%
12	20.23	19.32	20.01	99%	19.24	100%	19.68	97%	18.43	95%	19.58	97%	18.41	95%
13	21.00	20.13	20.85	99%	20.11	100%	20.51	98%	18.86	94%	20.43	97%	19.19	95%
14	21.92	20.70	—	—	—	—	—	—	—	—	21.20	97%	19.92	96%
15	22.78	21.50	—	—	—	—	—	—	—	—	21.98	96%	20.82	97%
16	23.52	22.40	—	—	—	—	—	—	—	—	22.80	97%	21.54	96%
17	22.98	21.72	—	—	—	—	—	—	—	—	22.38	97%	21.14	97%

Study average	15.64	14.63	15.49	99%	14.57	100%	15.10	97%	13.89	95%	15.08	96%	13.83	95%

Recovery average	22.80	21.58	—	—	—	—	—	—	—	—	22.09	97%	20.86	97%

%: percentage of control.

**Table 2:** Haematological findings (mean ± SD) in male rats after 90 days subchronic oral administration of Zigbir®.

Parameters	Control	250 mg/kg	500 mg/kg	1000 mg/kg	Control recovery	1000 mg/kg recovery
Hb (g%)	15.15 ± 1.45	15.39 ± 0.60	15.07 ± 0.59	14.89 ± 0.99	16.34 ± 1.60	16.50 ± 0.83
Total RBC (×10^6^/*μ*L)	7.00 ± 0.82	8.52** ± 0.60	7.50 ± 0.53	7.51 ± 0.50	8.35 ± 0.93	8.65 ± 0.34
Rt (%)	1.66 ± 0.42	1.59 ± 0.36	1.53 ± 0.41	1.60 ± 0.41	1.58 ± 0.41	1.66 ± 0.40
HCT (%)	38.38 ± 3.39	42.52** ± 1.77	40.65 ± 1.39	39.62 ± 2.47	45.58 ± 3.73	46.06 ± 2.30
MCV (*μ*m^3^)	55.01 ± 2.26	50.04** ± 2.23	54.33 ± 3.21	52.77 ±1.51	54.70 ± 2.40	53.26 ± 1.80
MCH (pg)	21.71 ± 0.98	18.09** ± 0.98	20.20** ± 1.29	19.81** ± 0.62	19.58 ±1.08	19.10 ± 0.75
MCHC (%)	39.48 ± 0.71	36.17** ± 0.54	37.15** ± 0.83	37.54** ± 0.36	35.80 ± 0.81	35.82 ± 0.33
Platelets (×10^3^/*μ*L)	396.90 ± 38.04	399.70 ± 67.67	394.40 ± 69.40	404.10 ± 84.93	453.00 ± 32.61	443.40 ± 66.39
Total WBC (×10^3^/*μ*L)	11.09 ± 2.94	12.52 ± 3.63	15.41* ± 4.79	13.53 ± 1.72	8.76 ± 2.06	10.84 ± 1.81
N (%)	21.60 ± 4.22	21.10 ± 2.92	20.90 ± 3.48	20.80 ± 4.49	20.40 ± 3.21	21.20 ± 3.11
L (%)	75.20 ± 3.99	75.90 ± 2.60	75.60 ± 3.57	76.20 ± 4.44	76.20 ± 2.95	75.80 ± 3.11
E (%)	1.50 ± 0.85	1.20 ± 0.79	1.10 ± 0.88	1.30 ± 0.82	1.20 ± 0.84	1.20 ± 0.84
M (%)	1.70 ± 0.95	1.80 ± 0.79	2.40 ± 0.84	1.70 ± 1.25	2.20 ± 0.84	1.80 ± 1.48
B (%)	0.00 ± 0.00	0.00 ± 0.00	0.00 ± 0.00	0.00 ± 0.00	0.00 ± 0.00	0.00 ± 0.00
Pt (s)	14.80 ± 2.44	14.60 ± 2.27	15.10 ± 2.77	15.30 ± 2.54	15.20 ± 2.39	15.60 ± 3.13

*P < 0.05 versus control group.

**P < 0.01 versus control group.

**Table 3:** Haematological findings (mean ± SD) in female rats after 90 days subchronic oral administration of Zigbir®.

Parameters	Control	250 mg/kg	500 mg/kg	1000 mg/kg	Control recovery	1000 mg/kg recovery
Hb (g%)	14.93 ± 0.83	14.79 ± 0.27	15.23 ± 0.79	15.19 ±1.10	16.74 ± 0.68	16.28 ± 0.33
Total RBC (×10^6^/*μ*L)	6.82 ± 0.94	7.63* ± 0.30	7.09 ± 0.31	6.99 ± 0.68	8.05 ± 0.49	7.58 ± 0.38
Rt (%)	1.61 ± 0.42	1.55 ± 0.40	1.80 ± 0.69	1.68 ± 0.44	1.60 ± 0.37	1.68 ± 0.26
HCT (%)	36.38 ± 3.25	39.78** ± 0.85	39.89** ± 1.95	38.98* ± 2.69	46.04 ± 1.51	44.10^#^ ± 0.97
MCV (*μ*m^3^)	53.61 ± 2.83	52.18 ± 1.46	56.27* ± 1.52	55.97 ± 2.90	57.28 ± 2.96	58.24 ± 2.20
MCH (pg)	22.11 ± 1.67	19.39** ± 0.63	21.48 ± 0.63	21.83 ± 1.02	20.88 ± 1.42	21.52 ± 0.89
MCHC (%)	41.22 ± 1.81	37.17** ± 0.62	38.16** ± 0.31	39.00** ± 0.33	36.38 ± 0.73	36.94 ± 0.47
Platelets (×10^3^/*μ*L)	482.80 ± 86.51	470.70 ± 69.16	432.60 ± 84.13	401.40 ± 67.02	467.20 ± 37.77	425.60 ± 48.19
Total WBC (×10^3^/*μ*L)	10.68 ± 3.72	8.92 ± 2.46	10.28 ± 3.75	8.53 ± 3.43	8.38 ± 2.11	8.88 ± 2.82
N (%)	21.00 ± 3.83	21.20 ± 3.49	21.10 ± 3.48	21.10 ± 3.48	21.80 ± 4.32	21.20 ± 3.03
L (%)	75.40 ± 3.98	75.80 ± 3.52	75.60 ± 2.91	75.30 ± 2.87	75.20 ± 4.02	75.20 ± 3.19
E (%)	1.30 ± 0.95	1.20 ± 0.79	1.10 ± 0.74	1.40 ± 0.70	0.80 ± 0.84	1.20 ± 0.84
M (%)	2.30 ±1.06	1.80 ± 0.79	2.20 ± 0.79	2.20 ± 0.92	2.20 ± 1.10	2.40 ± 1.14
B (%)	0.00 ± 0.00	0.00 ± 0.00	0.00 ± 0.00	0.00 ± 0.00	0.00 ± 0.00	0.00 ± 0.00
Pt (s)	14.90 ± 2.33	14.50 ± 2.37	14.60 ± 2.55	15.00 ± 2.62	15.00 ± 2.92	15.80 ± 2.86

*P < 0.05 versus control group.

**P < 0.01 versus control group.

^#^ *P* < 0.05 versus recovery control group.

**Table 4:** Clinical chemistry data (mean ± SD) of male rats after oral administration of Zigbir® for 90 days.

Parameters	Control	250 mg/kg	500 mg/kg	1000 mg/kg	Control recovery	1000 mg/kg recovery
Total protein (g%)	7.55 ± 0.47	7.63 ± 0.48	7.66 ± 0.56	7.58 ± 0.51	7.47 ± 0.47	7.40 ± 0.28
Albumin (g%)	3.42 ± 0.45	3.29 ± 0.43	3.49 ± 0.39	3.57 ± 0.42	3.65 ± 0.22	3.58 ± 0.26
Bilirubin (mg%)	0.65 ± 0.07	0.68 ± 0.11	0.72 ± 0.08	0.69 ± 0.08	0.72 ± 0.08	0.66 ± 0.05
Blood sugar (mg%)	88.50 ± 10.28	92.10 ± 10.64	90.90 ± 9.16	88.90 ± 8.27	96.40 ± 7.09	97.40 ± 12.28
Triglycerides (mg%)	105.30 ± 8.56	102.10 ± 7.23	107.50 ± 7.43	108.20 ± 3.33	105.00 ± 4.64	107.20 ± 1.30
Cholesterol (mg%)	61.30 ± 5.68	58.60 ± 2.72	63.30 ± 6.50	64.20 ± 4.98	63.80 ± 4.02	62.20 ± 6.30
BUN (mg%)	37.20 ± 5.05	38.10 ± 7.14	41.10 ± 4.61	39.60 ± 6.36	40.20 ± 2.28	39.20 ± 4.44
Creatinine (mg%)	0.99 ± 0.14	0.98 ± 0.11	0.99 ± 0.11	0.98 ± 0.10	1.02 ± 0.10	1.04 ± 0.14
AST (IU/L)	62.00 ± 4.00	61.10 ± 4.58	60.90 ± 3.31	64.20 ± 4.76	61.80 ± 8.11	60.40 ± 6.19
ALT (IU/L)	43.80 ± 6.56	38.90 ± 7.88	40.40 ± 8.96	41.50 ± 6.08	38.80 ± 3.19	37.60 ± 6.31
ALP (IU/L)	68.80 ± 6.83	69.90 ± 5.99	70.80 ± 6.58	115.40** ± 12.40	73.00 ± 8.49	66.00 ± 5.10
*γ*GT (U/L)	14.10 ± 2.73	16.70 ± 4.50	14.80 ± 2.90	14.90 ± 3.28	18.80 ± 4.21	17.00 ± 1.58
LDH (IU/L)	358.30 ± 31.75	354.00 ± 26.99	345.30 ± 28.80	361.60 ± 28.48	357.40 ± 32.26	337.00 ± 33.59
CPK (IU/L)	63.40 ± 4.99	62.20 ± 4.96	67.20 ± 4.13	64.60 ± 3.57	63.40 ± 3.78	67.40 ± 2.97
Sodium (mmol/L)	141.30 ± 5.62	142.20 ± 6.58	141.20 ± 6.71	136.50 ± 5.66	138.60 ± 1.14	140.40 ± 6.15
Chloride (mmol/L)	101.60 ± 3.47	101.50 ± 2.92	101.80 ± 3.16	101.40 ± 2.88	102.20 ± 2.68	101.80 ± 2.77
Potassium (mmol/L)	3.70 ± 0.35	3.58 ± 0.15	3.66 ± 0.22	3.75 ± 0.23	3.81 ± 0.10	3.84 ± 0.06
Calcium (mg%)	9.64 ± 0.66	9.55 ± 0.51	9.63 ± 0.53	9.78 ± 0.74	9.96 ± 0.53	10.04 ± 0.72
Phosphorus (mg%)	4.30 ± 0.32	4.31 ± 0.42	4.06 ± 0.28	4.08 ± 0.43	4.12 ± 0.57	4.34 ± 0.05

**P < 0.01 versus control group.

**Table 5:** Clinical chemistry data (mean ± SD) of female rats after oral administration of Zigbir® for 90 days.

Parameters	Control	250 mg/kg	500 mg/kg	1000 mg/kg	Control recovery	1000 mg/kg recovery
Total protein (g%)	7.54 ± 0.38	7.54 ± 0.56	7.83 ± 0.53	7.55 ± 0.46	7.79 ± 0.35	7.64 ± 0.29
Albumin (g%)	3.40 ± 0.32	3.46 ± 0.43	3.18 ± 0.41	3.34 ± 0.48	3.29 ± 0.35	3.67 ± 0.25
Bilirubin (mg%)	0.68 ± 0.07	0.67 ± 0.10	0.73 ± 0.04	0.64 ± 0.08	0.64 ± 0.09	0.66 ± 0.06
Blood sugar (mg%)	92.10 ± 12.48	88.80 ± 13.95	93.80 ± 11.48	83.60 ± 8.57	91.80 ± 10.89	91.00 ± 7.28
Triglycerides (mg%)	104.70 ± 9.14	105.30 ± 9.04	102.50 ± 9.42	106.30 ± 7.79	102.40 ± 3.78	105.60 ± 3.21
Cholesterol (mg%)	63.20 ± 4.47	61.80 ± 6.61	64.50 ± 5.48	64.20 ± 4.98	66.20 ± 3.03	65.40 ± 5.94
BUN (mg%)	38.90 ± 4.82	41.60 ± 4.58	38.30 ± 4.97	36.80 ± 5.71	38.40 ± 7.37	41.40 ± 4.28
Creatinine (mg%)	0.98 ± 0.09	0.97 ± 0.12	0.98 ± 0.11	0.95 ± 0.07	0.97 ± 0.05	1.03 ± 0.10
AST(IU/L)	59.90 ± 5.51	61.20 ± 5.03	62.50 ± 4.55	63.50 ± 4.35	62.20 ± 8.32	65.80 ± 5.97
ALT (IU/L)	43.20 ± 6.36	42.40 ± 7.44	38.90 ± 7.99	39.50 ± 9.44	39.60 ± 6.80	37.60 ± 7.27
ALP (IU/L)	63.90 ± 7.06	66.90 ± 5.34	69.60 ± 8.28	108.80** ± 9.41	71.00 ± 9.08	71.60 ± 6.31
*γ*GT(U/L)	15.50 ± 2.92	14.80 ± 4.32	15.90 ± 2.81	15.90 ± 3.54	15.80 ± 2.39	15.60 ± 3.51
LDH (IU/L)	358.50 ± 33.58	354.50 ± 31.02	371.50 ±16.57	359.60 ± 34.88	334.60 ± 26.69	369.80 ± 34.49
CPK (IU/L)	63.70 ± 4.22	63.20 ± 5.01	64.50 ± 4.50	62.60 ± 4.79	66.40 ± 3.13	65.00 ± 5.66
Sodium (mmol/L)	142.10 ± 5.30	138.80 ± 5.69	141.80 ± 6.20	139.70 ± 6.31	139.40 ± 3.36	136.00 ± 5.10
Chloride (mmol/L)	101.90 ± 3.51	101.80 ± 2.82	101.40 ± 3.63	101.20 ± 2.94	102.40 ± 1.67	102.20 ± 1.79
Potassium (mmol/L)	3.73 ± 0.15	3.51* ± 0.14	3.83 ± 0.15	3.68 ± 0.24	3.77 ± 0.08	3.79 ± 0.06
Calcium (mg%)	9.44 ± 0.60	9.41 ± 0.50	9.49 ± 0.67	9.47 ± 0.70	9.52 ± 0.50	9.98 ± 0.68
Phosphorus (mg%)	4.23 ± 0.31	4.11 ± 0.27	4.21 ± 0.39	4.30 ± 0.37	4.34 ± 0.44	4.00 ± 0.50

*P < 0.05 versus control group.

**P < 0.01 versus control group.

**Table 6:** Absolute organ weights (g; mean ± SD) in male rats after 90 days subchronic oral administration of Zigbir®.

Organ	Control	250 mg/kg	500 mg/kg	1000 mg/kg	Control recovery	1000 mg/kg recovery
Terminal body weight	348.86 ± 18.31	342.77 ± 28.01	332.90 ± 24.41	339.11 ± 32.83	367.32 ± 29.61	347.08 ± 37.86
Brain	1.978 ± 0.056	1.958 ± 0.059	1.898 ± 0.114	1.961 ± 0.058	1.965 ± 0.165	2.003 ± 0.045
Liver	11.621 ± 1.218	10.544 ± 1.264	10.307 ± 2.068	10.314 ± 1.801	8.769 ± 1.679	9.520 ± 1.092
Kidneys	2.587 ± 0.245	2.498 ± 0.225	2.371 ± 0.300	2.507 ± 0.290	2.472 ± 0.348	2.570 ± 0.237
Adrenals	0.0516 ± 0.009	0.0593 ± 0.009	0.056 ± 0.005	0.0524 ± 0.009	0.0578 ± 0.009	0.0516 ± 0.008
Testes	2.893 ± 0.263	2.834 ± 0.270	3.000 ± 0.341	2.849 ± 0.235	2.647 ± 0.259	2.848 ± 0.401
Heart	1.128 ± 0.100	1.046 ± 0.084	0.996 ± 0.141	1.008 ± 0.121	0.994 ± 0.113	1.057 ± 0.057
Spleen	1.048 ± 0.214	1.126 ± 0.340	1.203 ± 0.351	1.176 ± 0.182	1.086 ± 0.301	1.190 ± 0.235
Lungs	1.606 ± 0.236	1.769 ± 0.217	1.585 ± 0.340	1.612 ± 0.154	1.491 ± 0.261	1.317 ± 0.569

**Table 7:** Absolute organ weights (g; mean ± SD) in female rats after 90 days subchronic oral administration of Zigbir®.

Organ	Control	250 mg/kg	500 mg/kg	1000 mg/kg	Control recovery	1000 mg/kg recovery
Terminal body weight	232.66 ± 11.33	228.66 ± 14.67	216.17 ± 10.90	204.97 ± 6.75	268.78 ± 20.25	255.00 ± 12.83
Brain	1.843 ± 0.075	1.909 ± 0.079	1.805 ± 0.076	1.719 ± 0.075	1.837 ± 0.060	1.824 ± 0.054
Liver	6.940 ± 1.060	8.049 ± 0.478	7.062 ± 0.898	6.111 ± 0.531	8.379 ± 1.137	7.635 ± 0.708
Kidneys	1.610 ± 0.147	1.745 ± 0.199	1.541 ± 0.133	1.459 ± 0.099	1.536 ± 0.218	1.615 ± 0.124
Adrenals	0.0666 ± 0.016	0.0747 ± 0.007	0.0676 ± 0.007	0.0628 ± 0.01	0.0608 ± 0.002	0.0712 ± 0.009
Ovaries	0.0956 ± 0.02	0.1077 ± 0.014	0.0942 ± 0.017	0.0956 ± 0.013	0.146 ± 0.043	0.1488 ± 0.029
Heart	0.764 ± 0.059	0.797 ± 0.039	0.750 ± 0.091	0.736 ± 0.079	0.828 ± 0.128	0.833 ± 0.089
Spleen	0.754 ± 0.163	0.802 ± 0.096	0.935 ± 0.195	0.725 ± 0.099	1.010 ± 0.063	0.868 ± 0.104
Lungs	1.307 ± 0.167	1.598 ± 0.428	1.514 ± 0.409	1.317 ± 0.168	1.007 ± 0.105	1.02 ± 0.149
Uterus	0.545 ± 0.155	0.549 ± 0.132	0.563 ± 0.167	0.492 ± 0.088	0.478 ± 0.119	0.522 ± 0.049

**Table 8:** Relative organ weights (% of fasting body weight; mean ± SD) in male rats treated with Zigbir® orally for 90 days.

Organ	Control	250 mg/kg	500 mg/kg	1000 mg/kg	Control recovery	1000 mg/kg recovery
Terminal body weight (g)	348.86 ± 18.31	342.77 ± 28.01	332.90 ± 24.41	339.11 ± 32.83	367.32 ± 29.61	347.08 ± 37.86
Brain	0.568 ± 0.027	0.574 ± 0.039	0.571 ± 0.027	0.583 ± 0.060	0.536 ± 0.042	0.582 ± 0.056
Liver	3.331 ± 0.303	3.077 ± 0.280	3.083 ± 0.536	3.033 ± 0.384	2.412 ± 0.568	2.792 ± 0.570
Kidneys	0.743 ± 0.076	0.730 ± 0.058	0.711 ± 0.063	0.739 ± 0.043	0.676 ± 0.107	0.752 ± 0.139
Adrenals	0.0148 ± 0.003	0.0174 ± 0.003	0.0169 ± 0.002	0.0155 ± 0.003	0.0159 ± 0.003	0.0151 ± 0.003
Testes	0.829 ± 0.063	0.830 ± 0.094	0.906 ± 0.124	0.849 ± 0.116	0.725 ± 0.103	0.835 ± 0.179
Heart	0.324 ± 0.026	0.306 ± 0.016	0.298 ± 0.028	0.298 ± 0.027	0.271 ± 0.031	0.308 ± 0.044
Spleen	0.301 ± 0.062	0.331 ± 0.105	0.359 ± 0.098	0.349 ± 0.063	0.300 ± 0.099	0.341 ± 0.040
Lungs	0.461 ± 0.068	0.518 ± 0.067	0.478 ± 0.102	0.479 ± 0.062	0.411 ± 0.095	0.386 ± 0.181

**Table 9:** Relative organ weights (% of fasting body weight; mean ± SD) in female rats treated with Zigbir® orally for 90 days.

Organ	Control	250 mg/kg	500 mg/kg	1000 mg/kg	Control recovery	1000 mg/kg recovery
Terminal body weight (g)	232.66 ± 11.33	228.66 ± 14.67	216.17 ± 10.90	204.97 ± 6.75	268.78 ± 20.25	255.00 ± 12.83
Brain	0.794 ± 0.056	0.836** ± 0.030	0.836 ± 0.049	0.838 ± 0.018	0.686 ± 0.050	0.716 ± 0.034
Liver	2.995 ± 0.519	3.528 ± 0.231	3.262 ± 0.336	2.979 ± 0.202	3.114 ± 0.304	3.001 ± 0.316
Kidneys	0.693 ± 0.068	0.763 ± 0.072	0.715 ± 0.081	0.712 ± 0.048	0.573 ± 0.081	0.634 ± 0.056
Adrenals	0.0285 ± 0.006	0.0327 ± 0.003	0.0314 ± 0.004	0.0307 ± 0.005	0.0228 ± 0.003	0.028^#^ ± 0.005
Ovaries	0.0411 ± 0.009	0.047 ± 0.005	0.0437 ± 0.008	0.0466 ± 0.006	0.0541 ± 0.014	0.0588 ± 0.014
Heart	0.329 ± 0.031	0.35 ± 0.028	0.347 ± 0.037	0.359 ± 0.033	0.307 ± 0.036	0.327 ± 0.033
Spleen	0.324 ± 0.071	0.351 ± 0.041	0.435** ± 0.105	0.354 ± 0.049	0.377 ± 0.028	0.341 ± 0.046
Lungs	0.563 ± 0.084	0.695 ±0.163	0.695 ± 0.159	0.642 ± 0.073	0.375 ± 0.037	0.399 ± 0.045
Uterus	0.236 ± 0.071	0.240 ± 0.053	0.263 ± 0.084	0.240 ± 0.043	0.176 ± 0.035	0.205 ± 0.024

**P < 0.01 versus control group.

^#^ *P* < 0.05 versus recovery control group.
